# The Learning Curve for Thermal Ablation of Liver Cancers: 4,363-Session Experience for a Single Central in 18 Years

**DOI:** 10.3389/fonc.2020.540239

**Published:** 2020-10-20

**Authors:** Xiang Jing, Yan Zhou, Jianmin Ding, Yijun Wang, Zhengyi Qin, Yandong Wang, Hongyu Zhou

**Affiliations:** ^1^ Department of Ultrasound, Tianjin Third Central Hospital, Tianjin, China; ^2^ Tianjin Institute of Hepatobiliary Disease, Tianjin Key Laboratory of Extracorporeal Life Support for Critical Diseases, Artificial Cell Engineering Technology Research Center, Tianjin Third Central Hospital, Tianjin, China; ^3^ Department of Hepatobiliary Surgery, Tianjin Third Central Hospital, Tianjin, China; ^4^ Department of Ultrasound, The Third Central Clinical College of Tianjin Medical University, Tianjin, China

**Keywords:** learning curve, thermal ablation, liver cancer, major complication, handing-down teaching

## Abstract

This study aimed to explore the special efforts required to achieve proficiency in performing thermal ablation of liver cancers, including tumors in difficult locations, and clarify the effects of handing-down teaching on the corresponding process. Major complications of patients receiving percutaneous thermal ablation of liver cancer were analyzed. Polynomial fitting was used to describe the connection between major complication rates and special experience. Learning curve of major complications was plotted both for the whole group and for each operator, respectively. Tumors in difficult locations were further studied. A total of 4,363 thermal ablation sessions were included in this study. 143 of 4,363 patients had major complications, corresponding to an incidence rate of 3.27%. 806 thermal ablation sessions were performed for tumors in difficult locations. The major complication rate of these sessions is 6.33%. According to the trend of the learning curve of the 4363 patients, the experience of the whole group can be classified into five stages, that is, the high-risk, relatively stable, unstable, proficient and stable periods. A learning curve for an individual operator can be classified into the high-risk, proficient and stable periods. The major complication rates for the chronologically first, second and third operator of the group are 3.23, 3.35, and 3.31%, respectively. The special experience needed to bypass the first stage corresponds to 410, 510, and 440 sessions, the second stage, 1850, 850, and 870 sessions, by the three operators, respectively. The major complication rates for the tumors in difficult locations for the first, second and third operator were 7.04, 5.53, and 5.98%, respectively. For the tumors in difficult locations, the special experience needed to bypass the first stage corresponds to 150, 130, and 140 sessions, the second stage, 290, 175, and 185 sessions, by the three operators, respectively. In conclusion, the learning process of an operator percutaneous thermal ablation for liver cancer can be classified into three stages. The major complication rate for tumors in difficult locations were higher than that for all tumors. Handing-down teaching can make an operator arrive at the third stage earlier but not the second stage.

## Introduction

Local thermal ablation techniques—including radiofrequency ablation, microwave ablation, laser ablation, and high intensity focused ultrasound (HIFU)—is widely used for the treatment of liver tumors in clinical practice (1–3). Among them, radiofrequency and microwave ablation are the most popular techniques (4, 5). Patients with liver cancer benefit significantly from the minimally invasive therapy. Previous studies show that the long-term outcome of patients treated by thermal ablation is comparable with that of surgical resection (6, 7). The major complications and perioperative mortality, however, were significantly lower in patients undergoing local thermal ablation (8, 9).

Major complication is a highly concerned evaluating indicator for thermal ablation. Although major complication may occur occasionally, an experienced operator, advanced equipment and use of assisting methods may help to significantly reduce the risk of major complication. Previous studies found that the rate of complication for thermal ablation ranges from 1.3 to 10.0% (10–13). With the increase of special experience and the development of equipment, the rate of complication will decrease. However, the major complication rates in different hospitals, countries, and areas are distinct (12–15). Therefore, similar to other minimally invasive treatments, thermal ablation for liver cancers is experience-dependent.

Thermal ablation is often regarded as a simple technique of inserting a needle to “burn” the tumor, without getting much attention to the details, assisting methods and skills. Despite minimal invasion of thermal ablation, its major complication is non-trivial and sometimes may lead to death (16, 17). Avoiding major complication by improving the special skill of the operators, therefore, is crucial. However, to our best knowledge, there is a lack of extensive study about the special efforts required to achieve proficiency in performing thermal ablation and reduce major complication.

A few studies have explored the learning process of the early period of thermal ablation (18–20). However, the number of patients enrolled in previous studies is small, which is far from sufficient to investigate the connection between special experience and possible major complication, with the rate of the latter being 2.0–4.0% (11, 13).

To bridge the aforementioned gap, in this paper, we studied the learning curve for thermal ablation of more than 4,000 sessions of liver cancers in our central. The effect of handing-down teaching on accelerating the learning process is clarified. Moreover, the special efforts required for treating tumors in difficult location are discussed.

## Materials and Methods

### Patients

The clinical data of patients undergoing thermal ablation for liver cancer from December 2001 to December 2019 were analyzed. The aim of all the thermal ablation is the radical treatment. The recommended indications for thermal ablation in our central were (1) patients having a solitary tumor with a size of ≤5 cm or multiple tumors (no more than 5) with a maximum size of ≤3 cm; (2) patients without portal vein tumor thrombus or extrahepatic metastasis; (3) patients with liver function of a Child-pugh classification A or B; (4) patients with a platelet count of ≥50×10^9^/L or INR ≤ 1.7. For patients that do not meet the aforementioned criteria, thermal ablation was decided on a case-by-case basis by the clinician. For patients with liver dysfunction or coagulation disorders, radical thermal ablation was performed after the liver function or coagulation function was improved. Patients (1) receiving thermal ablation for benign tumors, (2) receiving laparoscopic-assisted or open thermal ablation, or (3) undergoing thermal ablation combined with liver resection were excluded. A total of 4,363 patients with 4,363 percutaneous thermal ablation sessions were included in this study.

### Equipment

RFA procedures were performed using mono-polar RFA with cooled-shaft needles or umbrella electrodes without cooled-shaft needles (Mianyang Lide electronics co. LTD, Mianyang, China). The length of the electrodes ranged from 15 to 20 cm with a 2- or 3-cm active tip. The power was 200 W, and the frequency was 480 kHz. Cool-tip RFA system (Radionics, Burlington, MA, US) and RITA RFA system (Angio Dynamics. US) were adopted.

MWA procedures were carried out using an MTC-3 or MTC-3CA microwave therapy instrument (Forsea Microwave & Electronic Research Institute, Nanjing, China) with a frequency of 2,450 MHz. The MW antenna was a 14 G unipolar cooled-shaft needle with a 15-cm length and a 1.5-cm long active tip.

The ultrasound systems used for guidance were ALOKA5000 (Aloka, Tokyo, Japan), Philips iU22, Philips IU Elit, and Philips epic7 (Philips, Bothell, WA, USA), having a convex array probe with a frequency of 1.0–5.0 MHz.

### Ablation Procedures

Different ablation strategies were used depending on the size, morphology, and location of the tumor. Generally, for tumors ≤2 cm, single-point ablation was performed, whereas for tumors >2 cm, multi-point overlapping ablation was conducted. In addition, the safe margin for complete ablation of the tumor was 0.5 cm, unless the tumor was in a difficult location. Before 2005, the immediate efficacy was assessed based on the hyper-echoic region covering the tumor or the clinical experience of the operator. After 2005, CEUS was performed 15 to 20 min after thermal ablation to determine the immediate efficacy. For residual tumors determined by CEUS, supplementary ablation was performed. All the patients received contrast enhanced CT/MR to evaluate complete ablation one month after thermal ablation. All the treatments were performed by X. Jing, J. Ding, or Y. Wang individually. Among them, X. Jing was the first operator in our central, and J. Ding and Y. Wang was the second and third. The last two operators had more than 3-year experience of interventional ultrasound and more than 1,000 ultrasound guided procedures before doing thermal ablation. All the treatments were performed by free hand.

### Definition of Tumor Located in Difficult Locations

We defined a tumor in difficult location if the tumor is (1) within 5 mm from important tissues or organs (including diaphragm, gallbladder, biliary tract, large vessels, right kidney, and gastrointestinal tract), (2) within 5 mm from the liver capsule, and (3) an exophytic tumor.

### Ancillary Protocols for Tumors in Difficult Locations

(1) Artificial Ascites and Difficult Locations: For tumors adjacent to the extrahepatic tissues or organs, percutaneous puncture catheter drainage was conducted by inserting a 21 G or 18 G PTC needle into the adjoining site. If the adjoining site can restore water, then production of artificial ascites was undertaken. If the adjoining site cannot restore water, then the tissues or organs were prevented from thermal damage by dropping the ice saline solution continuously.
(2) Arterial Hydrothorax: For tumors adjacent to diaphragm or located at liver dome, percutaneous puncture catheter drainage was conducted by inserting an 18 G PTC needle into the pleural cavity. Then, 100–500 mL of ﬂuid was injected into the pleural cavity to obtain the safe and clear puncture path.
(3) Thermal Ablation Combined With PEIT: For tumors adjacent to the large vessels or biliary tract, a 21 G PTC needle was inserted into the side of the tumor close to the vessel or tract. Then, 1– 3 mL dehydrated alcohol was injected into the tumor. The injection of dehydrated alcohol and the thermal ablation were started at the same time.
(4) Tumor Blood Vessel Block: For exophytic tumors, the antenna or elector was inserted into the tumor blood vessel under the guidance of ultrasound or contrast enhanced ultrasound by passing through a portion of normal liver tissue. (If it cannot be performed, then the antenna was inserted into tumor directly). Then, the ablation was performed with a high power until the tumor presents as hypovascularity on CEUS.
(5) Thermal Ablation Combined With TACE: For tumors with a size of ≥5cm or with arteriovenous fistula, TACE was performed 1 or 2 weeks before thermal ablation. When the blood supply of the tumor was reduced and the patient was with liver function of a Child-pugh classification A or B, the radical thermal ablation was conducted.
(6) Image-Fusion and Navigation Systems: For lesions invisible on US and CEUS but detected by CECT or CEMRI, the antenna or elector was inserted under the guidance of US/CEUS-CECT/CEMRI fusing imaging.

It should be noted that in the early period of the development of our group, the same strategy (without assisting method or combined therapy) was performed for all the tumors, no matter whether they located in difficult location or not, because the aforementioned ancillary protocols were not established.

### Classification of Complications

Complications after thermal ablation were assessed according to the clinical symptoms, imaging findings and results of laboratory examinations. The definition of a major complication was a complication that requires further treatment, threatens the life of the patient, leads to substantial morbidity and disability, or results in a lengthened hospital stay (21). All other complications were considered to be minor.

#### Calculation of Learning Curve

The learning curves were calculated for the entire group and each operator, respectively. All the tumors and tumors in difficult locations were considered, respectively. The major complication rates were calculated based on moving averages of 50 samples. Polynomial fitting was used to describe the relationship between major complication rates and special experience. The periods of learning curve were identified based on the trend of the curve and the major complication rates. Complication rates of 4 and 2% were the cut-off values for identifying periods of the learning curves of all ablation sessions. Complication rates of 6 and 4% were the cut-off values for identifying periods of the learning curves of ablation sessions of tumors in difficult locations.

### Statistical Analysis

Continuous variables are expressed as the mean ± standard error and categorical variables as frequencies and percentages. The *x* ^2^-test or Fisher test was used to compare categorical data between different groups. For all tests, a *p* value < 0.05 was considered to indicate statistical significance. Statistical analyses were performed using the SPSS software (Version 17.0, IBM, Armonk, NY, USA).

## Results

A total of 4,363 patients undergoing percutaneous thermal ablation were included in this study. 143 of 4,363 patients had major complications with an incidence rate of 3.27% (**Table 1**). Six patients had a combined major complication. A total of 149 major complications occurred. Eight patients died during the periprocedural time (within 30 days of the thermal ablation) with a mortality of 0.18%. Among the eight patients, four of them died due to multiple organ failure, one died because of infectious shock, two died due to liver dysfunction, and one death happened by acute myocardial infarction after thermal ablation.

**Table 1:** Major complications after thermal ablation.

Complication	No. of complications
Hemorrhage	14
Intra-hepatic haematomas	3
Intra-peritoneal bleeding	6
Haemothorax	4
Subphrenic arterial hemorrhage	1
Bile duct injury	26
Biliary stenosis	8
Biloma combined with infection	11
Bile leak	5
Bronchobiliary fistula	2
Liver abscess	23
Diaphragmatic hernia	5
Liver dysfunction	5
Multiple organ failure	4
Intractable pleural effusion	49
Intractable ascites	10
Tumor implantation	6
Severe sepsis	4
Hepato-gastrointestinal fistula	1
gallbladder perforation	1
Massive arterioportal fistula	1

**Figure 1:**
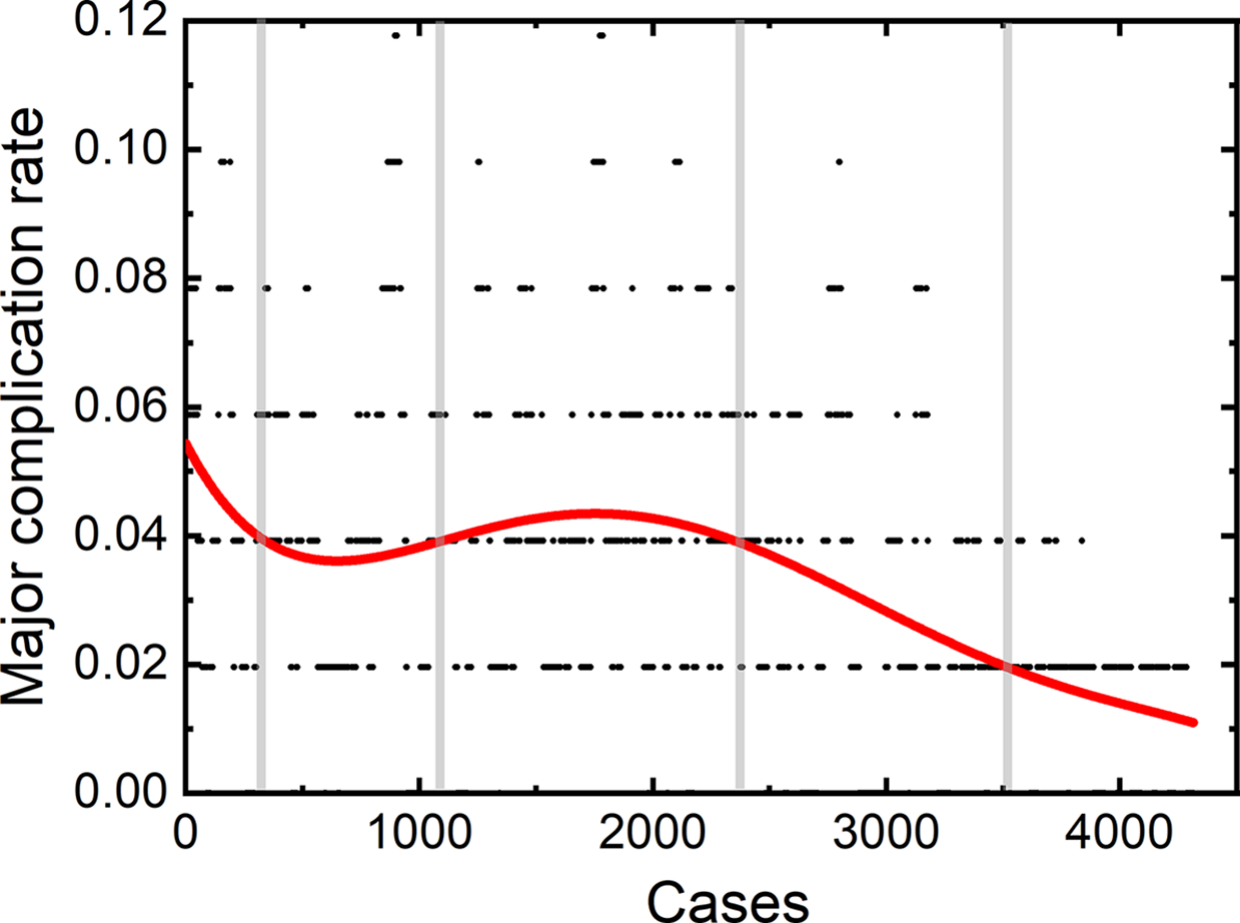
Learning curve for thermal ablation of all the 4,363 patients in our central.

The learning curve for thermal ablation of all the 4,363 patients in our central is shown in **Figure 1**. According to the trend of the learning curve, the experience of thermal ablation can be classified into five stages. The first stage was from the first patients to the 350^th^ patient, called high-risk period. The second stage was from the 351^th^ patient to 1150^th^ patient, called relative stable period. The third stage, named unstable period, was from the 1151^th^ patient to 2400^th^ patient. The fourth and fifth stage was coined proficient period and stable period, from the 2401^th^ patient to 3500^th^ patient and 3501^th^ patient to 4,363^th^ patient, respectively.

Three operators participated in treating the aforementioned patients. X. Jing was the first operator in our central. He started the first thermal ablation since December 2001 with an experience of the technique for 18 years. J. Ding was the second operator and started his first thermal ablation from the 928^th^ patient in our hospital with an experience of 8 years. The third operator was Y. Wang, who starts his first thermal ablation from the 1227^th^ patient, with an experience of 7.5 year for thermal ablation.

A total of 2,170 thermal ablation sessions were performed by the first operator. Among these sessions, 70 patients (3.23%) had major complications. 1,104 thermal ablation sessions were performed by the second operator with a major complication rate of 3.35% (37/1,104). The third operator achieved 1,089 sessions, with 36 (3.31%) major complications.

**Figure 2:**
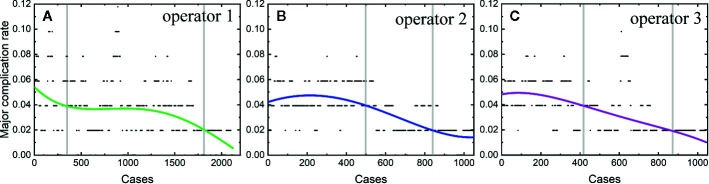
Learning curves of the operator 1 **(A)**, operator 2 **(B)** and operator 3 **(C)**.

The learning curves of each individual operator were depicted in **Figure 2**. The learning process can be classified into the high-risk, proficient and stable periods, according to the cut-off values of major complication rates of 4 and 2%. The experience needed to bypass the first stage corresponds to 410, 510, and 440 patients, and the second stage, 1,850, 850, and 870 patients, respectively.

**Figure 3:**
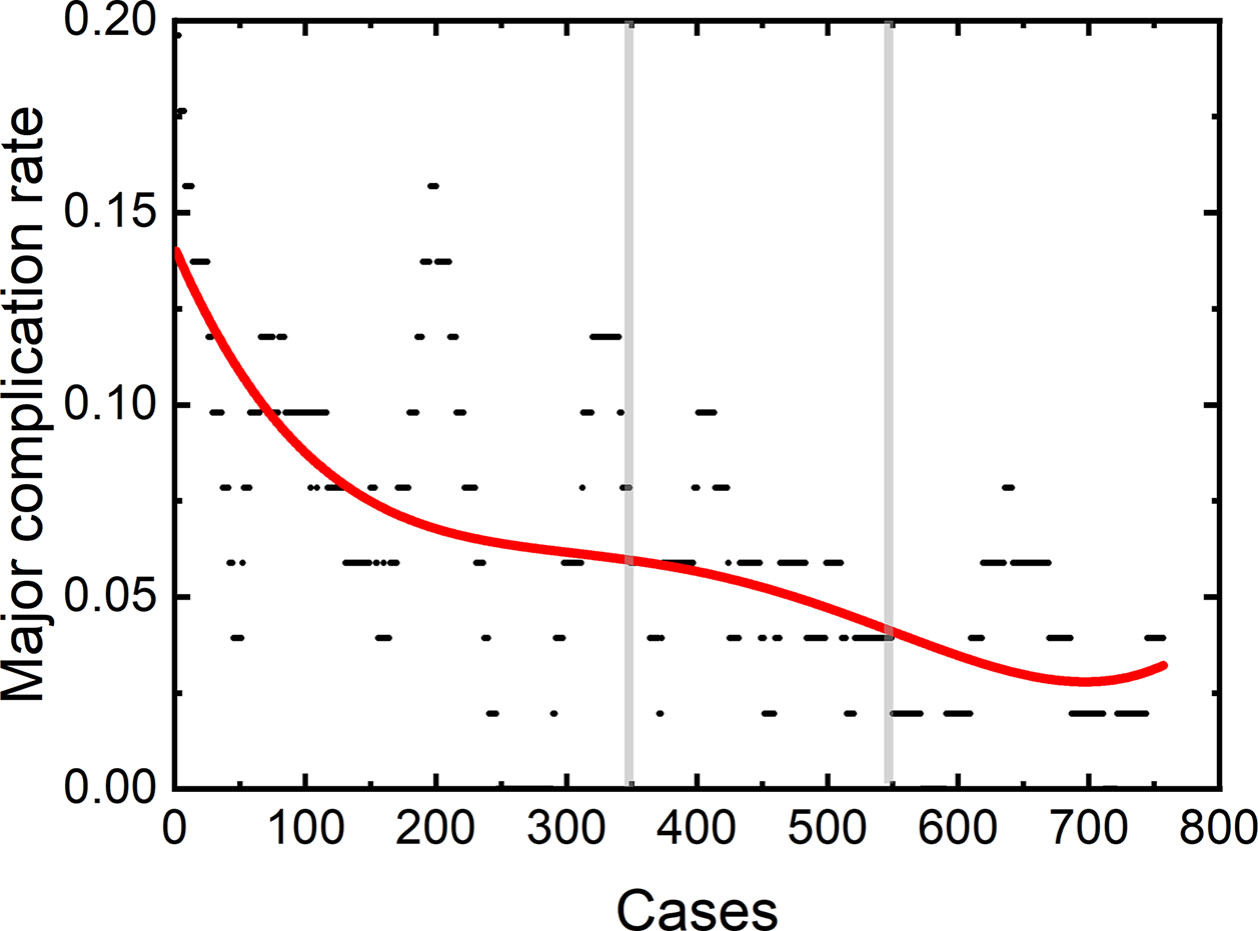
Learning curve for 806 sessions of tumors in difficult locations.

Among the 4,363 thermal ablation sessions, 806 sessions were performed for tumors in difficult locations with a proportion of 18.47%. 223 of the 806 sessions were with tumors adjacent to the large vessels or biliary tract (the first and second branch of biliary duct). Among the above 223 sessions, 159 ablation sessions were assisted with PEI. 57 of the 806 sessions were with tumors close to gallbladder. Hydro-dissection technique was used in 39 of the 57 sessions. 462 of the 806 sessions were with tumors located under liver capsule, including 351 sessions with tumors adjacent to diaphragm and 201 sessions with exophytic tumors. Among the 462 sessions, artificial ascites technique and arterial hydrothorax technique were used in 221 and 123 sessions, respectively. 61 of the 806 sessions were with tumors close to gastrointestinal tract. Artificial ascites technique was used in 45 of the 61 sessions. Artificial ascites technique was used in 3 sessions with tumors close to right kidney. The incidence rate of major complications in patients with a tumor in a difficult location was 6.33% (51/806). The rest 3,557 sessions correspond to 92 major complications with an incidence rate of 2.59%. The learning curve for thermal ablation of tumors in difficult locations was shown in **Figure 3**.

**Figure 4:**
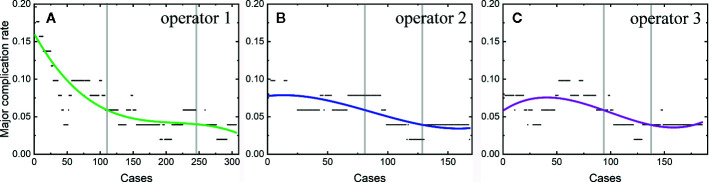
Learning curve for thermal ablation of tumors in difficult locations of the operator 1 **(A)**, operator 2 **(B)** and operator 3 **(C)**.

Among the 806 difficult sessions, 355 ones were performed by the first operator, with a major complication rate of 7.04%. 217 and 234 sessions were achieved by the second and third operator, respectively. 12 of 217 and 14 of 234 sessions correspond to major complications, with complication rates of 5.53 and 5.98%, respectively. The second and third operator started the thermal ablation for tumors in the difficult location from the 104^th^ and 141^th^ patients, respectively. According to the major complication rates at the cut-off values of 6 and 4%, a learning curve for thermal ablation of tumors in difficult locations of an individual operator was classified into the high-risk, proficient, and stable periods (**Figure 4**). The experience required to bypass the first stage corresponds to 150, 130, and 140 patients and the second, 290, 175, and 185, for the three operators, respectively.

## Discussion

The major complication rate of our central for the 4,363 radical thermal ablations of liver cancers was 3.27%, similar to those reported in previous studies (9, 22). According to the learning curve of individual operators in our hospital, the learning process can be classified into three stages, namely, the high-risk, proficient, and stable periods. The experience required to bypass the first period corresponds to similar number of patients for the three operators, which was not affected by handing-down teaching. However, handing-down teaching can significantly reduce experience needed to bypass the second period, as mirrored by the length of this period of the three operators. The peak value of the complication rate for thermal ablation of tumors in difficult locations is also reduced due to the handing-down teaching.

Recently, thermal ablation is widely used for primary and metastatic liver tumors and is popular with clinician due to its minimal invasion and safety (23, 24). Besides, thermal ablation is easy to learn, so that clinician in different professions started to use this therapy to treat liver cancer. In China, thermal ablation can be performed by clinician in the departments of interventional ultrasound, interventional radiography, and hepatobiliary surgery etc. However, the learning process of this technique does not attract the same attention as laparoscopic liver resection, robotic-assisted laparoscopic colorectal surgery, and laparoscopic cholecystectomy (25–27). Previous studies mentioned that the complication rates range from 1.3 to 10% (10–13). Although the risk of complications can be reduced by improving our ablation skills, using assisted methods or developing equipment. Assisted methods and ablation strategy have been reported to achieve complete ablation and reduce major complications in previous studies (28–32). The learning process of this technique has not been well studied. In our study, the learning curve of this technique has been explored, and the influence of handing-down teaching on the learning process has been analyzed.

The learning curve of 4,363 thermal ablation sessions in our single central shows five stages, namely, the high-risk, relative stable, unstable, proficient, and stable periods. A peak value of major complication rate of 5.5% appeared in the first stage. Then, the major complication rates decrease. The stable period of our group, with a major complication rate of 2% similar to a previous study (11), is after the relative stable, unstable, and proficient period. 1,150 patients in the first stage were treated by the first operator only. The major complication rate shows a mild trend of increase in the third stage, which may be caused by the new participants of the second and third operators. The second and third operators started their individual thermal ablation from the 928^th^ and 1227^th^ patients, which corresponds to the third stage (1151^th^–2400^th^). After that, the major complication rate decreased rapidly for the following 1,000 patients and became stable at 2%, which indicates that all the operators were skillful enough to perform thermal ablation.

We further plotted the learning curve for thermal ablation of each operator. According to the trends of the curves and the complication rates, we classified the learning process into three stages, according to the cut-off values of major complication rates of 4 and 2%. The first stage is the high-risk period with a major complication rate higher than 4%, the second stage is the proficient period having a rate higher than 2%, and the third stage with a stable complication rate of 2% is called the stable period. The results of our study demonstrate that the experience corresponding to about 400 to 500 patients were needed to bypass the first period. Under the guidance of handing-down teaching, the experience needed in the first period did not decrease for the second and third operator. Different from the first period, the required experience reduced significantly in the second period by the handing-down teaching. The first operator arrived at the stable period when treating the 1800^th^ patient, while the experience corresponding to only half number of patients was needed for the second and third operator. This result indicates that the handing-down teaching has an important effect on learning thermal ablation.

We found that the risk of major complication highly depends on the location of tumors, in agreement with a previous study (28). Patients with tumors in difficult locations have a high major complication rate, in accordance with previous results (28, 31). The major complication was usually caused by (1) puncturing the non-tumor tissues by electrode or antenna, (2) thermal damage. It has been reported that patients with tumors close to biliary duct or with tumors adjacent to diaphragm have a higher major complication rate (28, 33). In our study, the major complication rate of patients with tumors in difficult locations is 2.5 times as large as that of patients without a tumor located in difficult locations. Therefore, understanding the learning process of difficult thermal ablation is very important. We calculated the learning curve of 806 difficult thermal ablation sessions. Our results demonstrate that the peak value of major complication rate in the early period of 806 difficult sessions was higher than that of all the sessions (about 14 vs. 5.5%). Different from the trend of learning curve for all the sessions, the major complication rate for thermal ablation for tumors in difficult locations decreases rapidly and becomes stable at 4%. The second and third operators started their difficult ablation from the 104^th^ and 141^th^ patient with a tumor located in difficult locations, respectively. Besides, the trend of the learning curve was not affected by the participant of the second and third operators. Without any fluctuations, the complication rate on the learning curve for thermal ablations of tumors in difficult locations decreases gradually.

The learning curves for thermal ablations of tumors in difficult locations of each operator were calculated. The learning process was classified into three stages, namely, the high-risk, proficient and stable periods, according to the trend of curve and the cut-offs of complication rates of 6 and 4%. The handing-down teaching also shows an effect on the second period but a negligible effect on the first period. The experience of thermal ablation needed to bypass the second period were significantly reduced under the guidance of the teacher (the first operator: 290, the second operator: 175, and the third operator: 185). Furthermore, the peak values of major complication rates for thermal ablations of tumors in difficult locations were lower for “students” (the second and third operators) compared with that for the “teacher” (the first operator). The peak values of major complication rates for the second and third operators were about 8 to 9% compared with 16% for the first operator. All the above results indicate that thermal ablation for liver cancer is experience-dependent. The handing-down teaching can shorten the learning process and reduce peak value of the major complication rate.

The learning process of thermal ablation for liver cancer presents three stages. The first stage, namely, the high-risk period was the early stage of learning. Both in the learning processes of the team or individual operators, the highest major complication rates appear in the first stage. The experience needed to bypass the first stage of the learning process for all the patients and for patients with tumors in difficult locations was shown in our study. The beginner should pay more attention to the treatment of patients in the first stage. We found that the number of patients relevant to the first stage was stable, which may be determined by this technology itself. Thermal ablation for liver cancer is a minimally invasive treatment and easy-to-learn to perform, which shows low dependence on experience. Besides, the dependence on experience was concealed in the early stage due to the large portion of easy cases enrolled. We thought that the first learning stage of thermal ablation only means “operator can do it” not “operator can achieve it”. After the first stage, more cases of patients with tumors in difficult locations were performed in the second stage, namely, proficient stage. The effects of handing-down teaching for thermal ablation are significant in the second stage. Less experience of thermal ablation was needed to bypass the second stage under handing-down teaching and to arrive at the stable period. Except for reducing the need of experience, handing-down teaching can also reduce the peak value of the major complication rate in patients with tumors in difficult locations. However, it should be pointed out that all the operators in our central had an experience of more than 1,000 ultrasound-guided procedures, and all the thermal ablation were performed with the free-hand technique, which may have effects on the learning process of thermal ablation. To our knowledge, thermal ablation was performed by two operators with puncture trestle in some centrals (28, 34). Thus, the learning curve of thermal ablation in different central may be distinct.

Thermal ablation is not only experience-dependent but also equipment- and assisting method-dependent. In recent years, various assisting methods have been developed to reduce the risk of major complication. For example, PEIT and PTCD with intraductal chilled saline perfusion were used for tumors adjacent to large vessels or biliary duct (35, 36). Electrode or antenna deployed parallel to vessels can be also used to avoid damage of large vessels (28). For large tumors with rich blood supply, TACE can be performed before thermal ablation to weaken the influence of “heat sink effect” (37). The invisible lesions on US or CEUS were regarded as a contraindication for percutaneous US-guided thermal ablation. Now such lesions can be ablated under the guidance of US/CEUS-CECT/CEMRI fusing imaging (38–40). The No-Touch technique is used by inserting multiples electrodes around the periphery of the tumor and activating them sequentially to perform ablation with a sufficient peritumoural margin and decrease the risk of needle-path tumor implantation by avoiding direct puncture of the tumor (41, 42). The major complications in our hospital show that some major complications may largely appear in a specific period of time, such as skin burn or diaphragm damage. Among the 143 major complications in our central, only one case of several skin burns happened in the early stage of thermal ablation. The MTC-3CA microwave therapy instrument was performed for this patient. Although a cooled-shaft needle was used, the ablation antenna works in parallel with water-cooled circle. We turned on the ablation energy but not water-cooled circle, which caused the skin burn of patients. After that, the manufacturer changed the ablation antenna working in series with water-cooled circle and the skin burn never happens in our hospital. Most of the diaphragm damage also happened in the early stage of thermal ablation for the lack of effective assisting method. When the artificial ascites or pleural effusion technique has been developed, the major complications about diaphragm significantly decreased during the ablation procedure by using such assisting methods. Therefore, advanced equipment and effective assisting methods have the same importance as the experience of thermal ablation for reducing the risk of major complications.

Some limitations present in our study. First, although all the treatments in our central aim to achieve the radical treatment outcome, we focused on the learning curve about the major complication of thermal ablation but not the complete ablation rate or prognosis in this study. Second, considering the similarities of the two types of thermal ablation technologies, i.e., microwave ablation and radiofrequency ablation, they were not analyzed respectively.

## Conclusion

The learning process of thermal ablation is classified into the high-risk, proficient and stable periods. The major complication rate of patients with tumors in difficult locations are higher than that of patients without a tumor in difficult locations. The major complication rate is stabilized at 2% for thermal ablation of all the tumors and 4% for thermal ablation of tumors in difficult locations. Handing-down teaching can reduce the experience needed to bypass the second period and reduce the peak value of major complication rate for patients with tumors in difficult locations. Our results can help the operators, especially beginners, achieve proficiency in an efficient fashion.

## Data Availability Statement

The datasets generated for this study are available on request to the corresponding author.

## Ethics Statement

The studies involving human participants were reviewed and approved by Tianjin Third Central Hospital Institutional Review Board. Written informed consent for participation was not required for this study in accordance with the national legislation and the institutional requirements.

## Author Contributions

YZ and XJ designed the study and wrote the manuscript. JD and ZQ collected data. HZ, YW, and YW supervised the findings of this study. All authors contributed to the article and approved the submitted version.

## Funding

This work was supported the Tianjin Science and Technology Commission (nos. 17ZXMFSY00050 and No.17YFZCSY01070).

## Conflict of Interest

The authors declare that the research was conducted in the absence of any commercial or financial relationships that could be construed as a potential conflict of interest.
